# Network oscillations imply the highest cognitive workload and lowest cognitive control during idea generation in open-ended creation tasks

**DOI:** 10.1038/s41598-021-03577-1

**Published:** 2021-12-20

**Authors:** Wenjun Jia, Frederic von Wegner, Mengting Zhao, Yong Zeng

**Affiliations:** 1 Concordia Institute for Information Systems Engineering, Gina Cody School of Engineering and Computer Science, Concordia University, Montreal, QC H3G 2W1 Canada; 2 School of Medical Sciences, University of New South Wales, Wallace Wurth Building, Kensington, NSW 2052 Australia

**Keywords:** Cognitive control, Decision, Problem solving, Reading

## Abstract

Design is a ubiquitous, complex, and open-ended creation behaviour that triggers creativity. The brain dynamics underlying design is unclear, since a design process consists of many basic cognitive behaviours, such as problem understanding, idea generation, idea analysis, idea evaluation, and idea evolution. In this present study, we simulated the design process in a loosely controlled setting, aiming to quantify the design-related cognitive workload and control, identify EEG-defined large-scale brain networks, and uncover their temporal dynamics. The effectiveness of this loosely controlled setting was tested through comparing the results with validated findings available in the literature. Task-related power (TRP) analysis of delta, theta, alpha and beta frequency bands revealed that idea generation was associated with the highest cognitive workload and lowest cognitive control, compared to other design activities in the experiment, including problem understanding, idea evaluation, and self-rating. EEG microstate analysis supported this finding as microstate class C, being negatively associated with the cognitive control network, was the most prevalent in idea generation. Furthermore, EEG microstate sequence analysis demonstrated that idea generation was consistently associated with the shortest temporal correlation times concerning finite entropy rate, autoinformation function, and Hurst exponent. This finding suggests that during idea generation the interplay of functional brain networks is less restricted and the brain has more degrees of freedom in choosing the next network configuration than during other design activities. Taken together, the TRP and EEG microstate results lead to the conclusion that idea generation is associated with the highest cognitive workload and lowest cognitive control during open-ended creation task.

## Introduction

Design is a ubiquitous, complex, and open-ended creation behaviour^1^. All of us design consciously or unconsciously in everything we do. Societies design laws to govern social behaviours, engineers design products to achieve certain functions, artists design different forms to realize or release subconscious emotional energy, and economists design market mechanisms to enable trade. Over the past decades, design is emerging as a transdisciplinary domain in parallel with the arts and sciences. A lot of research has been endeavoring to model design as a rational problem-solving process^2^, a ‘reflective conversation with the situation’^3^, or a process involving both cognitive and affective components^4^, in which problems are actively framed by designers who take actions improving the current situation, approaching to satisfying solutions.

According to Bloom’s taxonomy^5^, behaviours related to design creation implicate various cognitive functions, such as problem understanding, idea generation, idea evaluation, idea elaboration, idea evolution, and ideation. Neuroimaging studies revealed that ideation is associated with heightened activation in the dorsolateral and medial prefrontal cortex^6–8^ and temporal brain regions^9^. Idea generation is related to activity in the default-mode network (DMN) while idea evaluation is linked to the executive control network^10–13^. EEG studies indicated that idea generation is associated with decreases in alpha power over almost all sites, suggesting increased cognitive workload^14–19^. An increase in cognitive workload frequently occurs at the beginning and end of idea generation^20,21^, an effect that influences the subjective rating of task difficulty^22^. Compared to idea generation, increases in alpha power are associated with idea elaboration and idea evolution^17,23^. In addition, idea evaluation is associated with increased theta power over mid-frontal sites, reflecting heightened cognitive control^24–28^. As such, these cognitive functions are related to large-scale brain networks during design creation.

Ongoing cognition during design activities is better characterized by the complex interaction of large-scale brain networks over time, rather than by an activation sequence of circumscribed brain areas reflecting basic cognitive functions^29–31^. These ongoing cognitive functions constitute the continuous flow of thought, varying with internally and externally changing experimental conditions. Yet, a quantitative description of how these large-scale brain networks temporally and functionally interact during design creation is largely missing.

Major challenges in studying design creation are the experimental paradigm and adequate analysis strategies with the presence of non-stationary data properties related to a free flowing task execution, as opposed to stimulus-averaged signals. A strictly controlled experiment^32^ that observes stimulus-response effects in the brain, whereas controlling all extraneous variables cannot simulate some key cognitive functions related to creativity during design creation, such as reframing and mind wandering^17^. In addition, the strictly controlled experiment itself will become a critical independent factor to influence the design results through changing a designer’s perception of the design problem due to design recursivity. Design recursivity demonstrates that the design problem, solution, and knowledge keep evolving interdependently and simultaneously^33–38^, which stresses the concept ‘an agent’s view of a world changes depending on what the agent does’^39^. Design recursivity results in the non-repetitiveness of the design process in that whatever designers do, and whatever changes in the environment will all contribute to updating the initial design problem. This non-repetitiveness defies repetition/averaging-based cognitive study techniques.

Therefore, we simulated design creation under loosely controlled settings in a series of open-ended creation tasks, including problem understanding, idea generation, idea evaluation, and self-rating. The loosely controlled setting refers to considerable freedom regarding response time (self-paced) and response action (integrating thinking and drawing phases) while maintaining certain degrees of control^40^. The effectiveness of loosely controlled settings was demonstrated in a recent creativity study^17^. To align our findings with other validated evidence, we investigated the regional contribution of brain oscillations in the classical frequency bands (delta, theta, alpha, and beta) to the different open-ended creation tasks through TRP analysis. In terms of data analysis, however, loosely controlled settings add new challenges. Several cognitive functions can be simultaneously involved in one open-ended creation task, and one cognitive function may contribute to different open-ended creation tasks. Basic cognitive functions never appear in isolation and interact heavily with each other. Consequently, causal relationships between stimuli and responses are extremely complex under loosely controlled settings.

To facilitate the loosely controlled experimental setting, we used EEG microstate analysis to segment the unstructured EEG signals into a set of microstates. Each microstate reflects activity in large-scale brain networks whose induced scalp potential fields remain quasi-stable during successive short time periods^41^. EEG microstates are closely associated with resting-state networks as identified using fMRI^42^ and cognitive components^43,44^, potentially representing the basic building blocks of consciousness, sometimes called “atoms of thoughts”^45–47^. Temporal dynamics of EEG microstate sequences increasingly attract the attention of scholars, reflecting the need to quantify the temporal structure and complexity of microstate sequences, running in parallel to, and thus representing the ongoing flow of thoughts and cognitive processes^48–50^. A few studies revealed key characterises of temporal dynamics underlying resting EEG microstate sequences, such as scale-free dynamics^51^, short- and long-range correlations^52^, and non-Markovianity^53^. In contrast to resting-state EEG microstate sequences, temporal dynamics of EEG microstate sequences are still unknown for design creation, which will be uncovered in this study.

We hypothesized that idea generation would be associated with the highest cognitive workload but the lowest cognitive control. It is during idea generation that participants are minimally constrained by instructions, and have the most freedom to explore and express their ideas, suggesting a relaxed cognitive control. The reduced cognitive control, however, may lead to increases in cognitive workload due to increased uncertainty coming from the relative freedom associated with this task. Therefore, one could expect: (1) idea generation would be associated with more decreases in delta, theta, alpha, and beta power^17^; (2) EEG microstate class C would be more prevalent during idea generation, since EEG microstate class C reflects activities in the default mode network^48,49^ and is associated with neural processes requiring less cognitive control, such as mind wandering^54^; and (3) the correlation time of microstate sequences to depend on cognitive control mechanisms. Stronger cognitive control would lead to more restricted microstate transitions and therefore longer correlation times. Correspondingly, idea generation would be associated with fewer restrictions in choosing the next brain state and lead to shorter microstate sequence correlation times.

## Methods

### Experiment design

**Figure 1:**
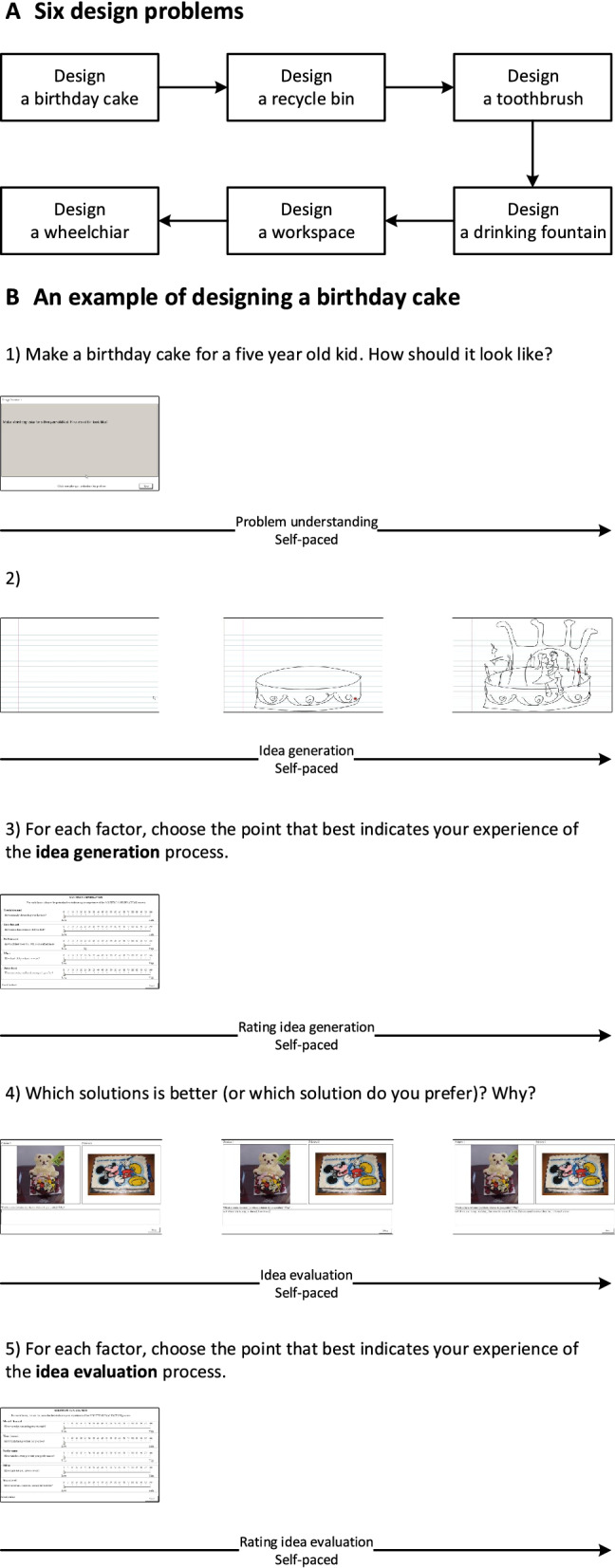
(**A**) The sequence of six design problems. (**B**) An example of schematic time courses of designing a birthday cake. (1) Self-paced problem understanding. (2) Self-paced idea generation. (3) Self-paced rating idea generation. (4) Self-paced idea evaluation. (5) Self-paced rating idea evaluation.

The experiment included six runs corresponding to six different design problems: a birthday cake, a recycle bin, a toothbrush, a wheelchair, a workspace, and a drinking fountain^22^, as listed in Table 1. Figure 1A shows the sequence of six design problems presented to the participants. Each run included five successive design activities: problem understanding, idea generation, rating idea generation, idea evaluation, and rating idea evaluation. Figure 1B shows an example of schematic time courses of designing a birthday cake.

**Table 1:** Six design problems.

Design problem	Description
Design a birthday cake	Make a birthday cake for a 5 year old kid. How should it look like?
Design a recycle bin	Sometimes, we do not know which items should be recycled. Create a recycle bin that helps people recycle correctly
Design a toothbrush	Create a toothbrush that incorporates toothpaste (incorporate = include, combine)
Design a wheelchair	In Montreal, people on wheelchair cannot use metro safely because most of metros have only stairs or escalators. Elevator is not an option because it is too costly to build one. You are asked to create the most efficient solution to solve this problem
Design a workspace	Employees in an IT company are sitting too much. The company wants their employees to stay healthy and work efficiently at the same time. You are asked to create a workspace that can help the employee to work and exercise at the same time
Design a drinking fountain	Two problems with standard drinking fountain: (a) filling up water bottle is not easy; (b) people too short cannot use the fountain and people too tall has to bend over too much. Create a new drinking fountain that solves these problems

During problem understanding, participants were asked to read and understand a given design problem. During idea generation, participants were asked to sketch a solution that satisfied the requirements of the design problem. During idea evaluation, participants were asked to evaluate the performance or preference of two given solutions. NASA Task Load Index was placed at the end of idea generation and evaluation, namely rating idea generation and rating idea evaluation respectively. During rating activity, participants were asked to rate their mental demand, time demand, performance, effort, and stress level. The rests were placed at the beginning and end of this experiment with eye-closed for three minutes. By dividing the experiment into the above mentioned five design activities, we aimed at a reduced difficulty in investigating such a complex design process by adding certain ‘structure’ to the unstructured data. Such segmentation was the main control applied in the experiment, meaning that despite the task description showing on the screen from the beginning of each design activity, any additional control including time limit, oral instructions, or ‘think-aloud’ related control, was avoided during the experiment. In the meanwhile, the presented experiment was loosely controlled as participants were given unlimited response time during each design activity and they were given sufficient freedom to complete the given task in their own way without any interruption or interference. In this way, the characteristics of design process could be better modelled as sufficient time and freedom could be essential to allow participants’ naturally exploring possible solutions and completing the given task recursively.

### Participants and experiment procedure

A total of 42 participants took part in this experiment, who were graduate students in the Gina Cody School of Engineering and Computer Science, Concordia University. A gift card of CAD$100 was given as compensation to the best design. Three participants were excluded from data analysis since they have not completed all the experiments. Eleven participants were excluded from data analysis due to technical errors such as missing markers. One participant was excluded from data analysis due to large electrode impedances and poor data quality. The final samples included 27 participants (8 women, 19 men) aged from 24 to 39. All participants had normal or corrected-to-normal vision. The experimenters helped subjects wear the HRV chest strap, GSR finger strap, respiration rate belt and EEG cap. The experimenters briefed each participant the experimental tasks; impedances of all the EEG electrodes were below 10 k$$\Omega$$; participants completed the experiment by following the experimental procedures specified in the experimental design. EEG signals were recorded by a 64 channel BrainVision actiCHamp at 500 Hz during the experiment. The EEG was referenced to Cz and the electrode placement was based on the international 10-10 system. The experimental protocol was approved by Human Research Ethics Committee (HREC) of Concordia University. All sections of the experiment were performed in accordance with relevant guidelines and regulations. All subjects signed the informed consent form before taking the experiment.

### Data analysis approach

#### Data pre-processing

The EEGLAB toolbox was used to reduce noise and remove artefacts from the EEG signals^55^. After acquisition, A one-pass zero-phase Hamming windowed-sinc FIR filter between 1 and 40 Hz was applied to the EEG signals. Second, bad global channels of the EEG signals were detected and isolated when one or more criteria were satisfied: a channel was flat for more than 5 s; a correlation between a channel and its nearby channels is smaller than 0.8; and a channel’s amplitude was greater than 3 standard deviations from the mean. Third, eye-blink, eye-movement, muscle-generated, and other artefacts were removed using the multiple artifact rejection algorithm (MARA) when independent components had more than 40$\%$ chance to be labelled as artefacts. Fourth, the EEG signals were segmented into 2-s epochs, with the aim to detect bad segments and bad local channels within segments^56^. Bad local channels in each segment were detected using FASTER^57^ criteria (variance, median gradient, amplitude range, and deviation from mean amplitude) when one or more Z scores of four criteria were greater than 3 standard deviations from the mean. The detected bad local channels were interpolated using spherical splines. Next, bad segments were rejected when one or more criteria were satisfied: a channel’ amplitude was higher than ±100 μV; the single electrode probability across segments or the electrode group probability within segments was greater than 3 standard deviations from the mean. Finally, the isolated bad global channels were interpolated using spherical splines. The cleaned EEG signals were re-referenced to average reference and were downsampled to 250 Hz.

#### Task-related power analysis

The power spectral density ($\mathrm {\mu V^2/Hz}$) of EEG signals was estimated by Welch’s method with a time window of 500 sample points and 250 sample points overlap between neighbouring time windows. The power ($\mathrm {Pow, \mu V^2}$) of EEG signals was estimated by the composite trapezoidal rule in the delta band (1–3.5 Hz), theta band (4–7.5 Hz), alpha band (8–13.5 Hz), and beta band (14–29 Hz)^58^. The task-related power (TRP) was computed in each channel (i) based on the formula $\mathrm {TRP_i = Log(Pow_{i, activation}) - Log(Pow_{i, reference})}$. The estimated log-power during the first resting state was subtracted from the estimated log-power during each design activities^59^. The condition-wise TRP was computed by averaging TRP across runs of the same condition. Positive TRP values reflect power increases from the rest to the design activities, whereas negative TRP values reflect power decreases from the rest to the design activities.

To compare with the previous studies, the condition-wise TRP values at 63 electrodes were grouped into the five cortical areas (frontal, central, temporal, parietal, and occipital) and two hemispheres (left and right)^18^. In the left hemisphere, the areas were defined as follows, frontal: Fp1, AF3, AF7, F1, F3, F5, F7, FC1, FC3; central: FC5, C1, C3, C5; temporal: FT7, T7, TP7, CP5, P5; parietal: CP1, CP3, P1, P3; and occipital: PO3, PO7, P7, O1. In the right hemisphere, the corresponding even-numbered electrodes were included.

Statistical analyses were performed on the design activities considering the selected five cortical areas in each hemisphere. The TRP changes were analyzed by a $\mathrm {5 \times 5 \times 2}$ repeated measures ANOVA with the three within factors CONDITION (problem understanding, idea generation, rating idea generation, idea evaluation, and rating idea evaluation), AREA (frontal, central, temporal, parietal, and occipital), and HEMISPHERE (left and right). Post-hoc comparisons of the TRP changes at AREA were performed with the Bonferroni correction between CONDITION. The Greenhouse-Geisser correction was applied in the case of sphericity violations.

#### EEG microstate analysis

The modified k-means clustering algorithm was applied to identify the microstate classes^60^. Firstly, for each run, each participant, and each condition, the Global Field Power (GFP) of EEG signals was calculated based on Eq. (1). The GFP is the standard deviation of the potentials across all electrodes at a given time point. Secondly, the EEG signals at GFP peaks were submitted to the modified k-means algorithm, which was run 100 times for cluster number $k=2\ldots 10$. Thirdly, the microstate classes were determined by minimizing the cost function defined in Eq. (2). The optimal microstate classes were selected among 100 repetitions based on minimum cross-validation as shown in Eq. (3). Fourthly, the group-wise microstate classes were determined by the full permutation procedure applied to the subject-wise microstate map across runs, participants, and conditions^61^.

1 $$\begin{aligned} GFP= & {} \sqrt{\frac{\sum _{i=i}^{N_S} (u_i-{\overline{u}})^2 }{N_S}}, \end{aligned}$$

2 $$\begin{aligned} F= & {} \frac{1}{N_T(N_S-1)}\sum _{t=1}^{N_T} \left |\left|V_t - \sum _{k=1}^{N_K}a_{kt}\Gamma _k \right |\right|^2, \end{aligned}$$

3 $$\begin{aligned} CV= & {} \frac{\sum _{t=1}^{N_T} (V_t'\cdot V_t - (V_t'\cdot \Gamma _k)^2)}{N_T(N_S-1)}\cdot \left( \frac{N_S-1}{N_S-1-N_K}\right) ^2, \end{aligned}$$

In Eqs. (1)–(3), $u_i$ is the electric potential of the EEG signals *u* at the electrode *i*, ${\overline{u}}$ is the average electric potential of all electrodes of the EEG signals *u* and $N_S$ is the number of electrodes of the EEG signals *u*. $N_T$ is the sample length . $V_t$ is a $N_S \times 1$ vector consisting of the electric potential at time instant *t*. $N_K$ is the number of microstate classes. $\Gamma _k$, which is a normalized $N_S \times 1$ vector, represents the k-th microstate class. $a_{kt}$ is the intensity of the k-th microstate class at the time instant *t*. Once the global microstate classes are determined, they were fitted back to the individual EEG signals in the time domain to generate the microstate sequences. Each time point of individual EEG signals was assigned into one of the global microstate classes when they have the highest spatial correlations. In the fitting process, the polarity of global microstate classes was ignored since the same neural generators may result in the inversion of scalp potential field. To avoid modifications of temporal dynamics of microstate sequences, we did not apply any criteria to smooth the microstate sequences, such as the minimum duration of microstates. For each run, for each participant, for each condition, and for each microstate class, the following microstate parameters were calculated:

- mean microstate duration: the average lifespan or duration that a microstate remains stable. The microstate duration can be interpreted as the average amount of time that a set of neural generators remains synchronously active.
- mean microstate occurrence: the average number of times that a microstate occurs per second. The mean microstate occurrence can be interpreted as the average amount of times that a set of neural generators becomes synchronously active.
- mean microstate coverage: the fraction of the total analysis time covered by a microstate. The microstate coverage can be interpreted as the relative rather than absolute presence of a microstate.

#### EEG microstate sequence analysis

A finite estimate of the entropy rate^62^, autoinformation function (AIF)^53^, and Hurst exponent estimated by detrended fluctuation analysis (DFA)^63^ were applied to measure the short-range, intermediate-range, and long-range temporal dependencies of microstate sequences within each run of conditions.

Firstly, the finite estimate of the entropy rate of microstate sequences was calculated based on Eq. (4)

4 $$\begin{aligned} \begin{aligned} h_{X}(t,k)&= H(X_{t+1}|X_{t}^{(k)}) \\&=H(X_{t+1}, X_t^{(k)}) - H(X_{t}^{(k)}) \\&=H(X_{t+1}^{(k+1)}) - H(X_{t}^{(k)}) \end{aligned} \end{aligned}$$

where $X_{t+1}$ represents the next symbol of the microstate sequences, while $X_{t}^{(k)}$ represents the past *k* values of the microstate sequences. $H(\cdot )$ represents joint entropy, while $H(\cdot |\cdot )$ represents conditional entropy. We used the logarithm to the base 2 for all entropy calculations, resulting in a unit of bits per sample for entropy rates.

Secondly, the AIF of microstate sequences was calculated based on Eq. (5).

5 $$\begin{aligned} \begin{aligned} I(\tau )&= H(X_{t+\tau }) - H(X_{t+\tau }|X_t) \\&= H(X_{t+\tau }) + H(X_{t}) - H(X_{t}, X_{t+\tau }) \end{aligned} \end{aligned}$$

Thirdly, the Hurst exponent of microstate sequences was estimated by DFA. Microstate sequences were first mapped into the metric space $S_0=\{-1, +1\}$^51^. As we used 7 microstate classes, we used partitions into one set of 3, and another one containing 4 microstate classes ($\{\{A,B,C\}, \{D,E,F,G\}\}$, for instance). In total, we obtained 35 different partitions and analyzed the arithmetic average of their DFA estimated Hurst exponent. Each microstate belonging to the first component of the partition was mapped to − 1, microstates from the second component to + 1, to the effect that each microstate sequence was mapped to the $\{-1, +1\}$ state space.

Based on the mapped microstate sequence $x'$, the partially integrated sequence *y*(*t*) was calculated based on Eq. (6). Then, the partially integrated sequence *y*(*t*) was segmented into windows of various sizes $\triangle n$ that were logarithmically spaced on a scale between four samples and $N_T$ samples. In each segmentation with $\triangle n$ samples, the linear trend $y_{\triangle n}(t)$ of the integrated signal *y*(*t*) was estimated by a least-squares fit, while the mean-squared residual between *y*(*t*) and $y_{\triangle n}(t)$ was calculated based on Eq. (7). The fluctuation was calculated through averaging $F(\triangle n)$ across all identically sized windows. Thus, the Hurst exponent was estimated by the slope of the fluctuations across the various window lengths.

6 $$\begin{aligned} y(t)= & {} \sum _{m=1}^t{x'(m)- {\overline{x}}'}, t \in [1,N_T] \end{aligned}$$

7 $$\begin{aligned} F(\triangle n)= & {} \sqrt{\frac{1}{\triangle n}\sum _{t=1}^{\triangle n}[y(t)- y_{\triangle n}(t)]^2} \end{aligned}$$

## Results

### Behavioural results

In rating idea generation, self-rated mental demand was 49.01 (SE = 3.25); self-rated time demand was 39.79 (SE = 3.68); self-rated performance was 54.23 (SE = 3.08); self-rated effort was 42.38 (SE = 3.60); and self-rated stress was 41.84 (SE = 3.59). In rating idea evaluation, self-rated mental demand was 39.38 (SE = 3.13); self-rated time demand was 32.14 (SE = 3.60); self-rated performance was 61.66 (SE = 3.04); self-rated effort was 36.29 (SE = 3.22); and self-rated stress was 32.45 (SE = 3.82). Task completion time was 56.61 s (SE = 4.99) for problem understanding, 284.98 s (SE = 15.83) for idea generation, 32.67 s (SE = 1.32) for rating idea generation, 161.61 s (SE = 8.68) for idea evaluation, 29.12 s (SE = 1.35) for rating idea evaluation.

Post hoc paired t tests revealed significant decreases in self-rated mental demand ($p=0.000$), self-rated time demand ($p=0.001$), self-rated effort ($p=0.007$), and self-rated stress ($p=0.003$) from idea generation to idea evaluation, as well as significant increases in self-rated performance ($p=0.010$).

### Task-related power results

In the delta band, the $5 \times 5 \times 2$ repeated measures ANOVA revealed two significant main effects of CONDITION ($F(4,104)=38.288, p=0.000, \eta ^2=0.596$) and AREA ($F(2.749,71.474)=27.045, p=0.000, \eta ^2=0.510$), as well as three significant interaction effects of CONDITION $\times$ AREA ($F(5.330,138.584)=33.620, p=0.000, \eta ^2=0.564$), CONDITION $\times$ HEMISPHERE ($F(4,104)=5.1987, p=0.001, \eta ^2=0.166$), and CONDITION $\times$ AREA $\times$ HEMISPHERE ($F(5.688,147.887)=4.525, p=0.000, \eta ^2=0.148$).

**Figure 2:**
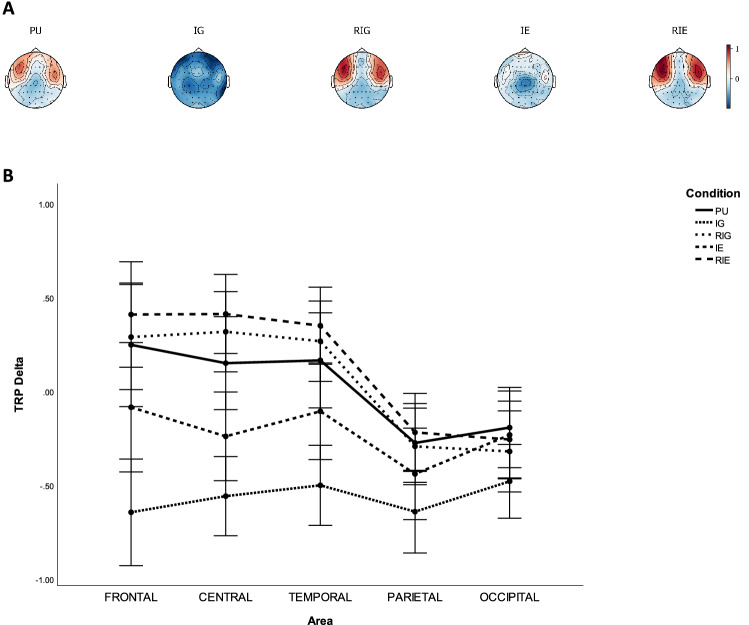
Task-related delta band power during problem understanding (PU), idea generation (IG), rating idea generation (RIG), idea evaluation (IE), and rating idea evaluation (RIE). (**a**) Grand average topographical maps of task-related delta power. (**b**) Error bars (SE) of task-related delta power.

Table 2 lists the p-values of pairwise comparisons of TRP delta with Bonferroni correction between CONDITION on each AREA, while Fig. 2 shows grand average topographical maps and error bars of task-related delta power between conditions. It was found that delta power was higher over frontal, central, temporal, parietal, occipital sites during PU compared to IG ($ps=0.000$), as well as over frontal and central sites during PU compared to during IE ($ps<0.046$), whereas it was lower over central sites during PU compared to during RIE. Delta power was lower over all sites during IG than during RIG, IE, and RIE ($ps<0.019$). Delta power was lower over frontal, central, temporal, and parietal sites during IE than during RIE ($ps<0.024$), as well as central and temporal sites during IE than during RIG ($ps<0.009$). In addition, it was found that delta power was larger over frontal sites of the left hemisphere compared to that of right hemisphere during PU and IG ($ps<0.022$). Delta power was larger over central sites of the left hemisphere compared to that of right hemisphere during RIG and RIE ($ps<0.013$). Delta power was larger over parietal sites of the right hemisphere compared to that of left hemisphere ($p=0.040$).

**Table 2:** P-values of pairwise comparisons with Bonferroni correction of TRP delta between AREA and CONDITION, including problem understanding (PU), idea generation (IG), rating idea generation (RIG), idea evaluation (IE), and rating idea evaluation (RIE).

Activity			Area									
			Frontal		Central		Temporal		Parietal		Occipital	
PU Vs. IG			0.0***	$\searrow$	0.0***	$\searrow$	0.0***	$\searrow$	0.000***	$\searrow$	0.0***	$\searrow$
PU Vs. RIG			1.0		0.499		1.0		1.0		0.498	
PU Vs. IE			0.046*	$\searrow$	0.005***	$\searrow$	0.69		0.271		1.0	
PU Vs. RIE			0.499		0.023*	$\nearrow$	0.1		1.0		1.0	
IG Vs. RIG			0.0***	$\nearrow$	0.0***	$\nearrow$	0.0***	$\nearrow$	0.000***	$\nearrow$	0.019*	$\nearrow$
IG Vs. IE			0.0***	$\nearrow$	0.000***	$\nearrow$	0.0***	$\nearrow$	0.004***	$\nearrow$	0.005***	$\nearrow$
IG Vs. RIE			0.0***	$\nearrow$	0.0***	$\nearrow$	0.0***	$\nearrow$	0.0***	$\nearrow$	0.001***	$\nearrow$
RIG Vs. IE			0.011		0.000***	$\searrow$	0.009**	$\searrow$	0.370		1.0	
RIG Vs. RIE			0.716		1.0		1.0		0.866		1.0	
IE Vs. RIE			0.001***	$\nearrow$	0.0***	$\nearrow$	0.001***	$\nearrow$	0.024*	$\nearrow$	1.0	

*$$\rho \le 0.050$$,   **$$\rho \le 0.010$$,   ***$$\rho \le 0.005$$.

$\nearrow$ TRP delta increases.

$\searrow$ TRP delta decreases.

In the theta band, the $5 \times 5 \times 2$ repeated measures ANOVA revealed two significant main effects of CONDITION ($F(4,104)=31.838, p=0.000, \eta ^2=0.550$) and AREA ($F(2.528,65.733)=25.767, p=0.000, \eta ^2=0.498$), as well as three significant interaction effects of CONDITION $\times$ AREA ($F(5.994,155.848)=26.820, p=0.000, \eta ^2=0.508$), CONDITION $\times$ HEMISPHERE ($F(4,104)=5.299, p=0.001, \eta ^2=0.169$), and CONDITION $\times$ AREA $\times$ HEMISPHERE ($F(6.340,164.833)=3.756, p=0.001, \eta ^2=0.126$).

**Figure 3:**
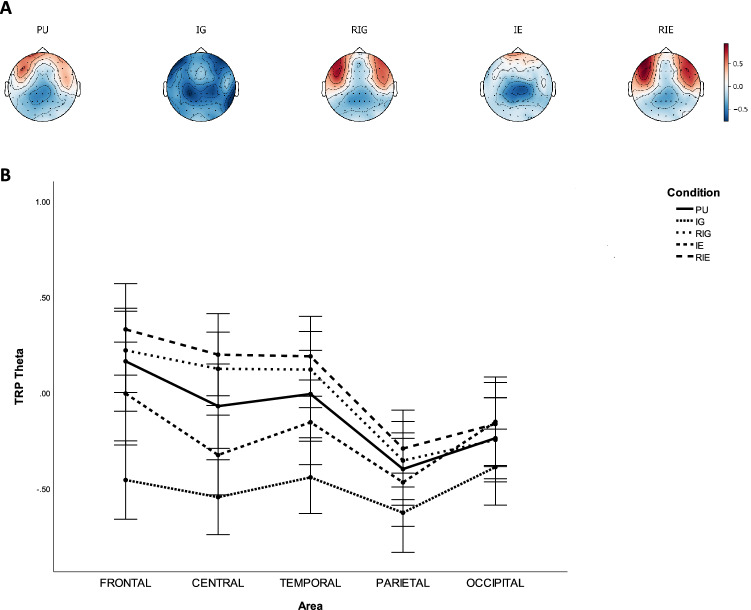
Task-related theta band power during problem understanding (PU), idea generation (IG), rating idea generation (RIG), idea evaluation (IE), and rating idea evaluation (RIE). (**a**) Grand average topographical maps of task-related theta power. (**b**) Error bars (SE) of task-related theta power.

Table 3 lists the p-values of pairwise comparisons of TRP theta with Bonferroni correction between CONDITION on each AREA, while Fig. 3 shows grand average topographical maps and error bars of task-related theta power between conditions. It was found that theta power was lower over frontal, central, temporal, parietal, and occipital sites during IG than during PU ($ps<0.036$), RIG ($ps<0.016$), IE ($ps<0.007$), and RIE ($ps<0.001$). Theta power was significantly lower over central sites during PU than during RIG ($p=0.015$), and over central and temporal sites during PU than during RIE ($ps<0.004$), whereas it was significantly higher over central sites during PU than during IE ($p=0.036$). Theta power was significantly lower over central and temporal sites during IE than during RIG ($ps<0.022$), as well as over frontal, central, temporal sites during IE than during RIE ($ps<0.005$). Besides, it was found that theta power was larger over frontal sites of the left hemisphere compared to that of right hemisphere during PU ($p=0.007$). Theta power was larger over central sites of the left hemisphere compared to that of right hemisphere during RIG and RIE ($ps<0.009$).

**Table 3:** P-values of pairwise comparisons with Bonferroni correction of TRP theta between AREA and CONDITION, including problem understanding (PU), idea generation (IG), rating idea generation (RIG), idea evaluation (IE), and rating idea evaluation (RIE).

Activity			Area									
			Frontal		Central		Temporal		Parietal		Occipital	
PU Vs. IG			0.0***	$\searrow$	0.0***	$\searrow$	0.0***	$\searrow$	0.002***	$\searrow$	0.036*	$\searrow$
PU Vs. RIG			1.0		0.015*	$\nearrow$	0.239		1.0		1.0	
PU Vs. IE			0.556		0.036*	$\searrow$	0.45		1.0		1.0	
PU Vs. RIE			0.067		0.001***	$\nearrow$	0.004***	$\nearrow$	0.12		0.608	
IG Vs. RIG			0.0***	$\nearrow$	0.0***	$\nearrow$	0.0***	$\nearrow$	0.001***	$\nearrow$	0.016*	$\nearrow$
IG Vs. IE			0.0***	$\nearrow$	0.001***	$\nearrow$	0.0***	$\nearrow$	0.007**	$\nearrow$	0.001***	$\nearrow$
IG Vs. RIE			0.0***	$\nearrow$	0.0***	$\nearrow$	0.0***	$\nearrow$	0.0***	$\nearrow$	0.001***	$\nearrow$
RIG Vs. IE			0.074		0.001***	$\searrow$	0.022*	$\searrow$	0.558		0.431	
RIG Vs. RIE			0.335		1.0		1.0		1.0		0.414	
IE Vs. RIE			0.005***	$\nearrow$	0.0***	$\nearrow$	0.002***	$\nearrow$	0.08		1.0	

*$$\rho \le 0.050$$,   **$$\rho \le 0.010$$,   ***$$\rho \le 0.005$$.

$\nearrow$ TRP theta increases.

$\searrow$ TRP theta decreases.

In the alpha band, the $5 \times 5 \times 2$ repeated measures ANOVA revealed two significant main effects of CONDITION ($F(4,104)=26.880, p=0.000, \eta ^2=0.508$) and AREA ($F(2.735,71.105)=51.801, p=0.000, \eta ^2=0.666$), as well as two significant interaction effects of CONDITION $\times$ AREA ($F(6.153,159.978)=21.723, p=0.000, \eta ^2=0.455$) and CONDITION $\times$ AREA $\times$ HEMISPHERE ($F(6.512,169.299)=3.700, p=0.001, \eta ^2=0.125$).

**Figure 4:**
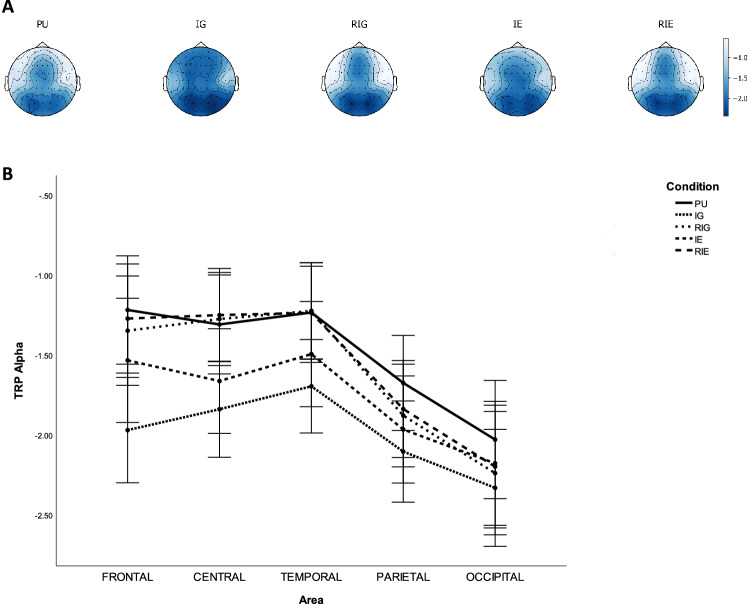
Task-related alpha band power during problem understanding (PU), idea generation (IG), rating idea generation (RIG), idea evaluation (IE), and rating idea evaluation (RIE). (**a**) Grand average topographical maps of task-related alpha power. (**b**) Error bars (SE) of task-related alpha power.

Table 4 lists the p-values of pairwise comparisons of TRP alpha with Bonferroni correction between CONDITION on each AREA, while Fig. 4 shows grand average topographical maps and error bars of task-related alpha power between conditions. It was found that decreases in alpha power were significantly smaller over frontal, central temporal, parietal, and occipital sites during PU compared to IG ($ps<0.001$), as well as over occipital sites during PU compared to RIG ($p=0.039$), as well as over frontal, central, temporal, and parietal sites during PU compared to IE ($ps<0.006$). Decreases in alpha power were significantly larger over frontal, central, temporal, and parietal sites during IG compared to during RIG ($ps<0.002$), as well as over frontal and temporal sites during IG compared to during IE ($ps<0.001$), as well as over frontal, central, temporal, parietal, and occipital sites during IG compared to during RIE ($ps<0.017$). In addition, decreases in alpha power were significantly larger over central and temporal sites during IE compared to during RIG ($ps<0.015$), as well as over frontal, central, and temporal sites during IE compared to RIE ($ps<0.015$).

**Table 4:** P-values of pairwise comparisons of TRP alpha with Bonferroni correction between AREA and CONDITION, including problem understanding (PU), idea generation (IG), rating idea generation (RIG), idea evaluation (IE), and rating idea evaluation (RIE).

Activity			Area									
			Frontal		Central		Temporal		Parietal		Occipital	
PU Vs. IG			0.0***	$\searrow$	0.0***	$\searrow$	0.0***	$\searrow$	0.001***	$\searrow$	0.001***	$\searrow$
PU Vs. RIG			0.297		1.0		1.0		0.246		0.039*	$\searrow$
PU Vs. IE			0.004***	$\searrow$	0.001***	$\searrow$	0.004***	$\searrow$	0.006**	$\searrow$	0.401	
PU Vs. RIE			1.0		1.0		1.0		0.484		0.102	
IG Vs. RIG			0.0***	$\nearrow$	0.0***	$\nearrow$	0.0***	$\nearrow$	0.002***	$\nearrow$	0.367	
IG Vs. IE			0.0***	$\nearrow$	0.052		0.01**	$\nearrow$	0.138		0.082	
IG Vs. RIE			0.0***	$\nearrow$	0.0***	$\nearrow$	0.0***	$\nearrow$	0.0***	$\nearrow$	0.017*	$\nearrow$
RIG Vs. IE			0.088		0.001***	$\searrow$	0.015*	$\searrow$	1.0		1.0	
RIG Vs. RIE			0.89		1.0		1.0		1.0		1.0	
IE Vs. RIE			0.015*	$\nearrow$	0.001***	$\nearrow$	0.01**	$\nearrow$	0.431		1.0	

*$$\rho \le 0.050$$,   **$$\rho \le 0.010$$,   ***$$\rho \le 0.005$$.

$\nearrow$ TRP alpha increases.

$\searrow$ TRP alpha decreases.

In the beta band, the $5 \times 5 \times 2$ repeated measures ANOVA revealed two significant main effects of CONDITION ($F(2.864,74.459)=41.769, p=0.000, \eta ^2=0.616$) and AREA ($F(4,104)=43.514, p=0.000, \eta ^2=0.626$), as well as two significant interaction effects of CONDITION $\times$ AREA ($F(6.372,165.660)=30.607, p=0.000, \eta ^2=0.541$) and CONDITION $\times$ AREA $\times$ HEMISPHERE ($F(6.241,162.265)=3.425, p=0.003, \eta ^2=0.116$).

**Figure 5:**
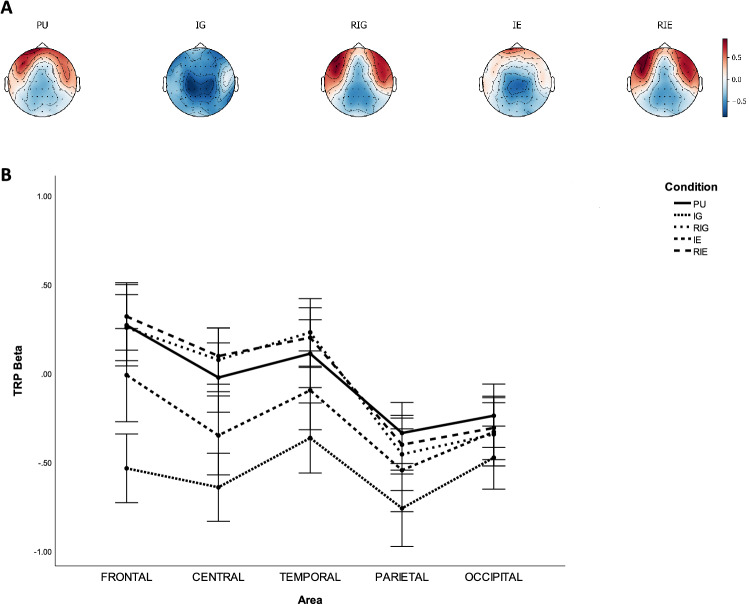
Task-related beta band power during problem understanding (PU), idea generation (IG), rating idea generation (RIG), idea evaluation (IE), and rating idea evaluation (RIE). (**a**) Grand average topographical maps of task-related beta power. (**b**) Error bars (SE) of task-related beta power.

Table 5 lists the p-values of pairwise comparisons of TRP beta with Bonferroni correction between CONDITION on each AREA, while Fig. 5 shows grand average topographical maps and error bars of task-related beta power between conditions. It was found that beta power increased significantly over frontal, central, temporal, parietal, and occipital sites from IG to PU ($ps<0.001$), RIG ($ps<0.041$), and RIE ($ps<0.001$), as well as over frontal, central, temporal and parietal sites from IG to IE ($ps<0.001$). The beta power increased significantly over frontal, central and temporal from IE to RIG ($ps<0.029$) and RIE ($ps<0.008$), as well as over frontal, central, temporal, and parietal sites from IE to PU ($ps<0.031$). Furthermore, it was found that beta power was larger over frontal sites of the left hemisphere compared to that of right hemisphere during PU ($p=0.009$).

**Table 5:** P-values of pairwise comparisons of TRP beta with Bonferroni correction between AREA and CONDITION, including problem understanding (PU), idea generation (IG), rating idea generation (RIG), idea evaluation (IE), and rating idea evaluation (RIE).

Activity			Area									
			Frontal		Central		Temporal		Parietal		Occipital	
PU Vs. IG			0.0***	$\searrow$	0.0***	$\searrow$	0.0***	$\searrow$	0.0***	$\searrow$	0.001***	$\searrow$
PU Vs. RIG			1.0		0.945		0.463		0.412		0.547	
PU Vs. IE			0.012*	$\searrow$	0.002***	$\searrow$	0.031*	$\searrow$	0.007**	$\searrow$	0.988	
PU Vs. RIE			1.0		0.264		0.711		1.0		0.751	
IG Vs. RIG			0.0***	$\nearrow$	0.0***	$\nearrow$	0.0***	$\nearrow$	0.0***	$\nearrow$	0.041*	$\nearrow$
IG Vs. IE			0.0***	$\nearrow$	0.001***	$\nearrow$	0.001***	$\nearrow$	0.001***	$\nearrow$	0.135	
IG Vs. RIE			0.0***	$\nearrow$	0.0***	$\nearrow$	0.0***	$\nearrow$	0.0***	$\nearrow$	0.001***	$\nearrow$
RIG Vs. IE			0.029*	$\searrow$	0.001***	$\searrow$	0.006**	$\searrow$	1.0		1.0	
RIG Vs. RIE			1.0		1.0		1.0		1.0		1.0	
IE Vs. RIE			0.008**	$\nearrow$	0.001***	$\nearrow$	0.007**	$\nearrow$	0.147		1.0	

*$$\rho \le 0.050$$,   **$$\rho \le 0.010$$,   ***$$\rho \le 0.005$$.

$\nearrow$ TRP beta increases.

$\searrow$ TRP beta decreases.

### EEG microstate results

#### EEG microstate classes

**Figure 6:**
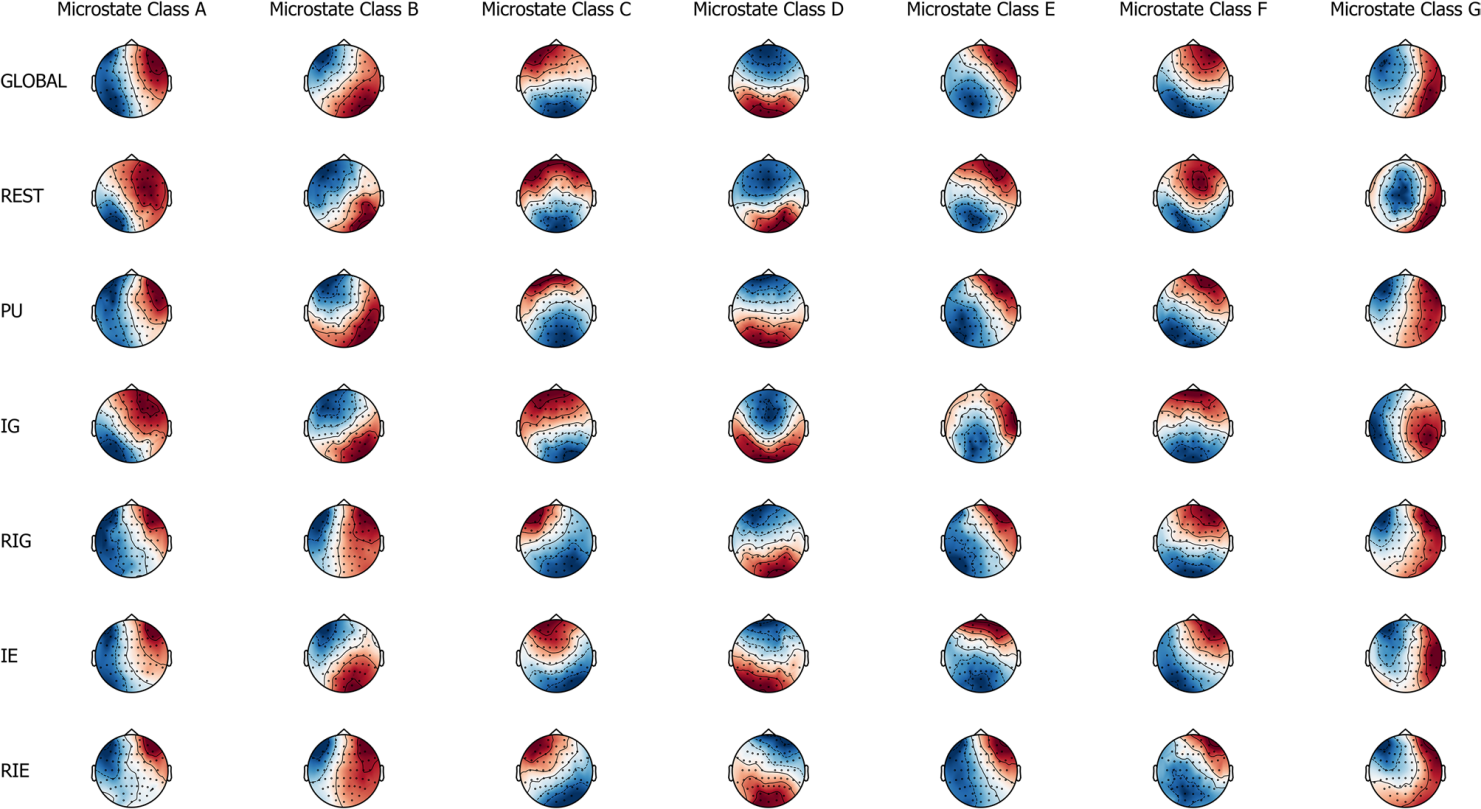
The spatial configuration of the seven microstate classes (A, B, C, D, E, F, and G) for across conditions (global) and within conditions (rest, problem understanding, idea generation, rating idea generation, idea evaluation, and rating idea evaluation).

Based on cross validation as shown in Eq. (3), the optimal number of individual microstate classes was found to be equal to 7.109 (SE = 0.134) for rest, 7.370 (SE = 0.079) for problem understanding, 7.547 (SE = 0.080) for idea generation, 7.849 (SE = 0.089) for rating idea generation, 7.458 (SE = 0.087) for idea evaluation, and 7.156 (SE = 0.302) for rating idea evaluation. Figure 6 shows the topographic maps of seven microstate classes across and within conditions, which include rest, problem understanding, idea generation, rating idea generation, idea evaluation, and rating idea evaluation. The seven microstate classes were labelled as A, B, C, D, E, F, and G according to the studies of Michel and Koenig^42^ and Custo et al.^64^. The number of individual microstate classes was defined as seven for each run, each condition and each participant because of comparability and simplicity of statistical analysis.

The seven individual microstate classes explained 68.6% (SE = 0.7) of the global variance of the original EEG topographies corresponding to peaks of GFP for rest, 67.1% (SE = 0.4) for problem understanding, 67.4% (SE = 0.3) for idea generation, 65.8% (SE = 0.4) for rating idea generation, 67.3% (SE = 0.4) for idea evaluation, and 66.4% (SE = 0.4) for the rating idea evaluation.

#### EEG microstate parameters

For EEG microstate coverage, the 6 $\times$ 7 repeated measures ANOVA revealed a significant main effect CLASS ($F(3.737,,97.159)=4.498, p=0.003, \eta ^2=0.147$) and a significant interaction effect CONDITION $\times$ CLASS ($F(7.378,191.837)=37.213, p=0.000, \eta ^2=0.589$).

**Figure 7:**
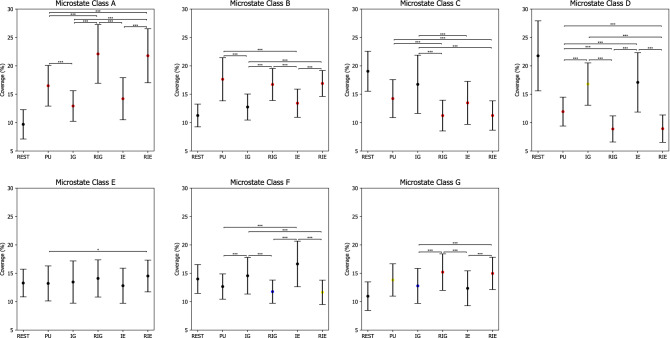
Microstate coverage during rest (REST), problem understanding (PU), idea generation (IG), rating idea generation (IE), idea evaluation (IE), and rating idea evaluation (RIE). P-values between rest and other conditions are annotated by black dots ($p>0.050$), blue dots ($p\le 0.050$), yellow dots ($p\le 0.010$), and red dots ($p\le 0.005$). P-values between conditions are annotated by * ($p\le 0.050$), ** ($p\le 0.010$), *** ($p\le 0.005$). Error bars: standard error of the mean.

Table 6 lists p-values of post hoc paired t tests with Bonferroni correction on microstate coverage, while Fig. 7 shows error bars of microstate coverage in each condition. In particular, the coverage of microstate class A was the lowest during REST compared to during PU, IG, RIG, IE, and RIE ($ps=0.000$), while the coverage of microstate class B was the lowest during REST compared to PU, RIG, IE, and RIE ($ps<0.005$). Similarly, the coverage of microstate class G was the lowest during REST compared to during PU, IG, RIG, and RIE ($ps<0.040$). On the contrary, the coverage of microstate class C was the highest during REST compared to during PU, RIG, IE, and RIE ($ps<0.001$), while the coverage of microstate class D was the highest during REST compared to PU, IG, RIG, and RIE ($ps<0.009$). The coverage of microstate class F was higher during REST compared to during RIG and RIE ($ps<0.021$).

In addition, the coverage of microstate class A decreased significantly from RIG and RIE to PU, IG, and IE ($ps<0.001$), as well as from PU to IG ($p=0.001$). The coverage of microstate class B increased significantly from IG and IE to PU, RIG, and RIE ($ps<0.001$). The coverage of microstate class C decreased significantly from IG to RIG, RIE, and IE ($ps<0.003$), as well as from PU to RIG and RIE ($ps<0.002$). The coverage of microstate class D decreased significantly from IG and IE to PU, RIG, and RIE ($ps<0.002$), as well as from PU to RIG and RIE ($ps<0.001$). The coverage of microstate class E increased significantly from PU to RIE ($p=0.028$). The coverage of microstate class F decreased significantly from IG and IE to PU, RIG, and RIE ($ps<0.003$). The coverage of microstate class G decreased significantly from RIG and RIE to IG and IE ($ps<0.002$).

For EEG microstate duration, the 6 $\times$ 7 repeated measures ANOVA revealed two significant main effects CONDITION ($F(3.099.80.585)=48.401, p=0.000, \eta ^2=0.651$) and CLASS ($F(3.616,94.017)=5.896, p=0.000, \eta ^2=0.185$), as well as a significant interaction effect CONDITION $\times$ CLASS ($F(6.911,179.685)=41.691, p=0.000, \eta ^2=0.616$).

**Table 6:** P-values of pairwise comparisons for microstate coverage with Bonferroni correction between CLASS (A, B, C, D, E, F, G) and CONDITION (rest (REST), problem understanding (PU), idea generation (IG), rating idea generation (RIG), idea evaluation (IE), and rating idea evaluation (RIE)).

Condition			Microstate classes													
			Class A		Class B		Class C		Class D		Class E		Class F		Class G	
REST Vs. PU			0.0***	$\nearrow$	0.0***	$\nearrow$	0.001***	$\searrow$	0.0***	$\searrow$	1.0		0.91		0.008**	$\nearrow$
REST Vs. IG			0.0***	$\nearrow$	0.064		0.734		0.009**	$\searrow$	1.0		1.0		0.04*	$\nearrow$
REST Vs. RIG			0.0***	$\nearrow$	0.0***	$\nearrow$	0.0***	$\searrow$	0.0***	$\searrow$	1.0		0.021*	$\searrow$	0.0***	$\nearrow$
REST Vs. IE			0.0***	$\nearrow$	0.005***	$\nearrow$	0.001***	$\searrow$	0.071		1.0		0.054		0.394	
REST Vs. RIE			0.0***	$\nearrow$	0.0***	$\nearrow$	0.0***	$\searrow$	0.0***	$\searrow$	1.0		0.009**	$\searrow$	0.0***	$\nearrow$
PU Vs. IG			0.001***	$\searrow$	0.0***	$\searrow$	0.056		0.0***	$\nearrow$	1.0		0.002***	$\nearrow$	0.962	
PU Vs. RIG			0.001***	$\nearrow$	1.0		0.002***	$\searrow$	0.001***	$\searrow$	0.881		0.272		0.379	
PU Vs. IE			0.223		0.001***	$\searrow$	1.0		0.002***	$\nearrow$	1.0		0.003***	$\nearrow$	0.271	
PU Vs. RIE			0.001***	$\nearrow$	1.0		0.001***	$\searrow$	0.001***	$\searrow$	0.028*	$\nearrow$	0.335		0.488	
IG Vs. RIG			0.0***	$\nearrow$	0.0***	$\nearrow$	0.0***	$\searrow$	0.0***	$\searrow$	1.0		0.001***	$\searrow$	0.001***	$\nearrow$
IG Vs. IE			0.492		1.0		0.003***	$\searrow$	1.0		1.0		0.628		1.0	
IG Vs. RIE			0.0***	$\nearrow$	0.0***	$\nearrow$	0.0***	$\searrow$	0.0***	$\searrow$	1.0		0.002***	$\searrow$	0.002***	$\nearrow$
RIG Vs. IE			0.0***	$\searrow$	0.001***	$\searrow$	0.1		0.0***	$\nearrow$	0.727		0.001***	$\nearrow$	0.002***	$\searrow$
RIG Vs. RIE			1.0		1.0		1.0		1.0		1.0		1.0		1.0	
IE Vs. RIE			0.0***	$\nearrow$	0.0***	$\nearrow$	0.055		0.0***	$\searrow$	0.388		0.001***	$\searrow$	0.001***	$\nearrow$

*$$\rho \le 0.050$$,   **$$\rho \le 0.010$$,   ***$$\rho \le 0.005$$.

$\nearrow$ microstate coverage increases.

$\searrow$ microstate coverage decreases.

**Figure 8:**
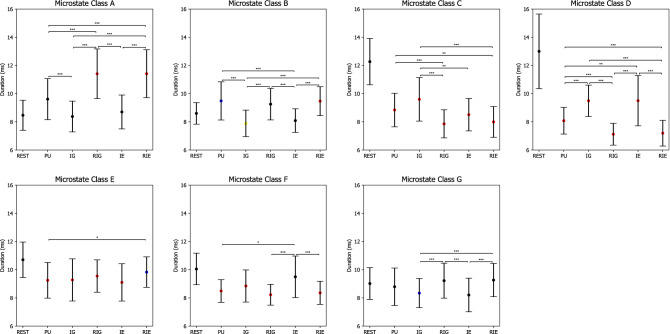
Microstate duration during rest (REST), problem understanding (PU), idea generation (IG), rating idea generation (IE), idea evaluation (IE), and rating idea evaluation (RIE). P-values between rest and other conditions are annotated by black dots ($p>0.050$), blue dots ($p\le 0.050$), yellow dots ($p\le 0.010$), and red dots ($p\le 0.005$). P-values between conditions are annotated by * ($p\le 0.050$), ** ($p\le 0.010$), *** ($p\le 0.005$). Error bars: standard error of the mean.

Table 7 lists p-values of post hoc paired t tests with Bonferroni correction on microstate duration, while Fig. 8 shows error bars of microstate duration in each condition. In particular, the duration of microstate class A was lower during REST compared to during RIG and RIE ($ps=0.000$). The duration of microstate class B was lower during REST compared to during PU ($p=0.044$), whereas it was higher during REST compared to during IG ($p=0.007$). The duration of microstate classes C, D and E was the lowest during REST compared to PU, IG, RIG, IE, and RIE ($ps<0.002$). The duration of microstate class F was higher during REST compared to during PU, IG, RIG, and RIE ($ps<0.004$). The duration of microstate class G was higher during REST compared to during IG ($p=0.027$).

**Table 7:** P-values of pairwise comparisons with Bonferroni correction for microstate duration between CLASS (A, B, C, D, E, F, G) and CONDITION (rest (REST), problem understanding (PU), idea generation (IG), rating idea generation (RIG), idea evaluation (IE), and rating idea evaluation (RIE)).

Condition			Microstate classes													
			Class A		Class B		Class C		Class D		Class E		Class F		Class G	
REST Vs. PU			0.079		0.044*	$\nearrow$	0.0***	$\searrow$	0.0***	$\searrow$	0.001***	$\searrow$	0.0***	$\searrow$	1.0	
REST Vs. IG			1.0		0.007**	$\searrow$	0.0***	$\searrow$	0.0***	$\searrow$	0.0***	$\searrow$	0.004***	$\searrow$	0.027*	$\searrow$
REST Vs. RIG			0.0***	$\nearrow$	0.113		0.0***	$\searrow$	0.0***	$\searrow$	0.001***	$\searrow$	0.0***	$\searrow$	1.0	
REST Vs. IE			1.0		0.123		0.0***	$\searrow$	0.0***	$\searrow$	0.001***	$\searrow$	1.0		0.069	
REST Vs. RIE			0.0***	$\nearrow$	0.005***	$\nearrow$	0.0***	$\searrow$	0.0***	$\searrow$	0.02*	$\searrow$	0.0***	$\searrow$	1.0	
PU Vs. IG			0.001***	$\searrow$	0.0***	$\searrow$	0.081		0.0***	$\nearrow$	1.0		0.409		0.419	
PU Vs. RIG			0.001***	$\nearrow$	1.0		0.001***	$\searrow$	0.001***	$\searrow$	1.0		0.787		0.918	
PU Vs. IE			0.062		0.001***	$\searrow$	1.0		0.01**	$\nearrow$	1.0		0.034*	$\nearrow$	0.108	
PU Vs. RIE			0.001***	$\nearrow$	1.0		0.007**	$\searrow$	0.002***	$\searrow$	0.018*	$\nearrow$	1.0		0.17	
IG Vs. RIG			0.0***	$\nearrow$	0.0***	$\nearrow$	0.0***	$\searrow$	0.0***	$\searrow$	1.0		0.077		0.001***	$\nearrow$
IG Vs. IE			1.0		1.0		0.006**	$\searrow$	1.0		1.0		0.741		1.0	
IG Vs. RIE			0.0***	$\nearrow$	0.0***	$\nearrow$	0.0***	$\searrow$	0.0***	$\searrow$	0.381		0.282		0.0***	$\nearrow$
RIG Vs. IE			0.0***	$\searrow$	0.001***	$\searrow$	0.455		0.0***	$\nearrow$	1.0		0.003***	$\nearrow$	0.001***	$\searrow$
RIG Vs. RIE			1.0		1.0		1.0		1.0		0.526		1.0		1.0	
IE Vs. RIE			0.0***	$\nearrow$	0.0***	$\nearrow$	1.0		0.0***	$\searrow$	0.209		0.004***	$\searrow$	0.0***	$\nearrow$

*$$\rho \le 0.050$$,   **$$\rho \le 0.010$$,   ***$$\rho \le 0.005$$.

$\nearrow$ microstate duration increases.

$\searrow$ microstate duration decreases.

Besides, the duration of microstate class A decreased significantly from RIG and RIE to PU, IG, and IE ($ps<0.001$), as well as from PU to IG ($p=0.001$). The duration of microstate class B increased significantly from IG and IE to PU, RIG, and RIE ($ps<0.001$). The duration of microstate class C decreased significantly from PU to RIG and RIE ($ps<0.007$), as well as from IG to RIG, IE, and RIE ($ps<0.006$). The duration of microstate class D decreased from PU to RIG and RIE ($ps<0.002$), as well as from IG and IE to PU, RIG, and RIE ($ps<0.001$). The duration of microstate class E decreased significantly from RIE to PU ($p=0.018$). The duration of microstate class F decreased significantly from IE to PU, RIG, and RIE ($ps<0.034$). The duration of microstate class G decreased significantly from RIG and RIE to IG and IE ($ps<0.001$).

For EEG microstate occurrence, the 6 $\times$ 7 repeated measures ANOVA revealed two significant main effects CONDITION ($F(3.393.88.230)=34.593, p=0.000, \eta ^2=0.571$) and CLASS ($F(3.707,96.380)=10.582, p=0.000, \eta ^2=0.289$), as well as a significant interaction effect CONDITION $\times$ CLASS ($F(7.618,198.068)=33.086, p=0.000, \eta ^2=0.560$).

**Figure 9:**
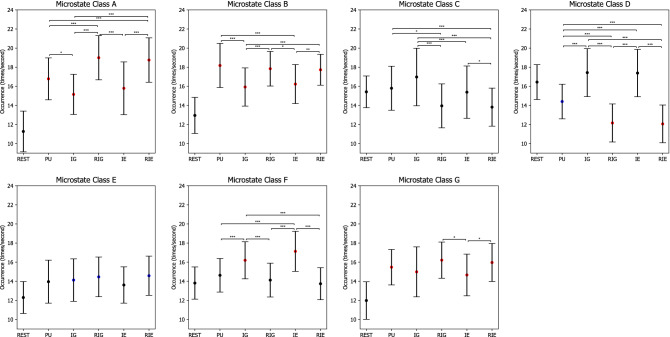
Microstate occurrence during rest (REST), problem understanding (PU), idea generation (IG), rating idea generation (IE), idea evaluation (IE), and rating idea evaluation (RIE). P-values between rest and other conditions are annotated by black dots ($p>0.050$), blue dots ($p\le 0.050$), yellow dots ($p\le 0.010$), and red dots ($p\le 0.005$). P-values between conditions are annotated by * ($p\le 0.050$), ** ($p\le 0.010$), *** ($p\le 0.005$). Error bars: standard error of the mean.

Table 8 lists p-values of post hoc paired t tests with Bonferroni correction on microstate occurrence, while Fig. 9 shows error bars of microstate occurrence in each condition. In particular, the occurrence of microstate classes A, B, and G increased significantly from REST to PU, IG, RIG, IE, and RIE ($ps=0.000$). Similarly, the occurrence of microstate class E increased significantly from REST to IG, RIG, and RIE ($ps<0.043$), while the occurrence of microstate class F increased significantly from REST to IG and IE ($ps=0.000$). On the contrary. the occurrence of microstate class D decreased significantly from REST to PU, RIG, and RIE ($ps<0.011$).

In addition, the occurrence of microstate class A decreased significantly from RIG and RIE to PU, IG, and IE ($ps<0.003$), as well as from PU to IG ($p=0.014$). The occurrence of microstate class B increased significantly from IG and IE to PU, RIG, and RIE ($ps<0.011$). The occurrence of microstate class C decreased significantly from PU to RIG and RIE ($ps<0.014$), from IG to RIG, IE, and RIE($ps<0.002$), as well as from IE to RIE ($p=0.016$). The occurrence of microstate class D decreased significantly from PU to RIG and RIE ($ps<0.001$), as well as from IG and IE to PU, RIG, and RIE ($ps<0.001$). The occurrence of microstate class F decreased significantly from IG and IE to PU, RIG, and RIE ($ps<0.001$). The occurrence of microstate class G increased significantly from IE to RIG and RIE ($ps<0.047$).

**Table 8:** P-values of pairwise comparisons with Bonferroni correction for microstate occurrence between CLASS (A, B, C, D, E, F, G) and CONDITION (rest (REST), problem understanding (PU), idea generation (IG), rating idea generation (RIG), idea evaluation (IE), and rating idea evaluation (RIE)).

Condition			Microstate classes													
			Class A		Class B		Class C		Class D		Class E		Class F		Class G	
REST Vs. PU			0.0***	$\nearrow$	0.0***	$\nearrow$	1.0		0.011*	$\searrow$	0.162		1.0		0.0***	$\nearrow$
REST Vs. IG			0.0***	$\nearrow$	0.0***	$\nearrow$	0.406		0.387		0.043*	$\nearrow$	0.0***	$\nearrow$	0.0***	$\nearrow$
REST Vs. RIG			0.0***	$\nearrow$	0.0***	$\nearrow$	0.332		0.0***	$\searrow$	0.015*	$\nearrow$	1.0		0.0***	$\nearrow$
REST Vs. IE			0.0***	$\nearrow$	0.0***	$\nearrow$	1.0		1.0		0.116		0.0***	$\nearrow$	0.0***	$\nearrow$
REST Vs. RIE			0.0***	$\nearrow$	0.0***	$\nearrow$	0.169		0.0***	$\searrow$	0.012*	$\nearrow$	1.0		0.0***	$\nearrow$
PU Vs. IG			0.014*	$\searrow$	0.0***	$\searrow$	0.229		0.001***	$\nearrow$	1.0		0.001***	$\nearrow$	1.0	
PU Vs. RIG			0.001***	$\nearrow$	1.0		0.014*	$\searrow$	0.001***	$\searrow$	1.0		1.0		0.462	
PU Vs. IE			0.999		0.003***	$\searrow$	1.0		0.001***	$\nearrow$	1.0		0.001***	$\nearrow$	0.997	
PU Vs. RIE			0.003***	$\nearrow$	1.0		0.002***	$\searrow$	0.001***	$\searrow$	0.335		0.404		1.0	
IG Vs. RIG			0.0***	$\nearrow$	0.001***	$\nearrow$	0.0***	$\searrow$	0.0***	$\searrow$	1.0		0.0***	$\searrow$	0.084	
IG Vs. IE			1.0		1.0		0.002***	$\searrow$	1.0		1.0		0.915		1.0	
IG Vs. RIE			0.0***	$\nearrow$	0.001***	$\nearrow$	0.0***	$\searrow$	0.0***	$\searrow$	1.0		0.001***	$\searrow$	0.378	
RIG Vs. IE			0.001***	$\searrow$	0.011*	$\searrow$	0.11		0.0***	$\nearrow$	0.554		0.001***	$\nearrow$	0.028*	$\searrow$
RIG Vs. RIE			1.0		1.0		1.0		1.0		1.0		1.0		1.0	
IE Vs. RIE			0.001***	$\nearrow$	0.006**	$\nearrow$	0.016*	$\searrow$	0.0***	$\searrow$	0.751		0.0***	$\searrow$	0.047*	$\nearrow$

*$$\rho \le 0.050$$,   **$$\rho \le 0.010$$,   ***$$\rho \le 0.005$$.

$\nearrow$ microstate occurrence increases.

$\searrow$ microstate occurrence decreases.

#### EEG microstate sequences

**Figure 10:**
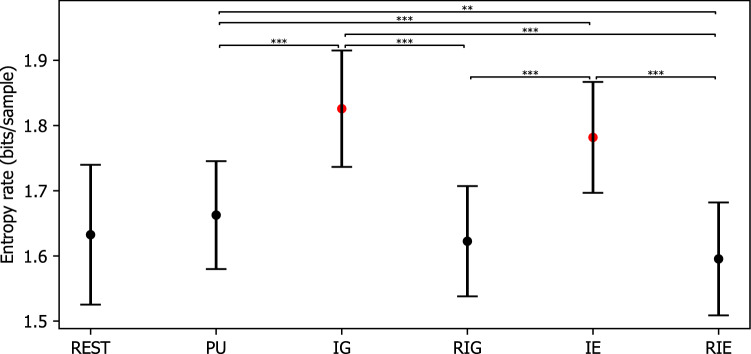
Entropy rate of microstate sequences during rest (REST), problem understanding (PU), idea generation (IG), rating idea generation (IE), idea evaluation (IE), and rating idea evaluation (RIE). P-values between rest and other conditions are annotated by black dots ($p>0.050$), blue dots ($p\le 0.050$), yellow dots ($p\le 0.010$), and red dots ($p\le 0.005$). P-values between conditions are annotated by * ($p\le 0.050$), ** ($p\le 0.010$), *** ($p\le 0.005$). Error bars: standard error of the mean.

The finite entropy rate was 1.633 bits/sample (SE = 0.021) for REST, 1.663 bits/sample (SE = 0.016) for PU, 1.826 bits/sample (SE = 0.018) for IG, 1.623 bits/sample (SE = 0.017) for RIG, 1.782 bits/sample (SE = 0.017) for IE, and 1.595 bits/sample (SE = 0.017) for RIE, when considering the previous 6 microstate labels. The repeated measures ANOVA revealed a significant effect CONDITION ($F(3.237,84.153)=40.629, p=0.000, \eta ^2=0.610$). Post hoc paired t tests with Bonferroni correction as shown in Fig. 10 indicated that the entropy rate was higher during IG and IE compared to during REST, PU, RIG, and RIE ($ps<0.001$), while the entropy rate was higher during PU compared to during RIE ($p=0.006$).

**Figure 11:**
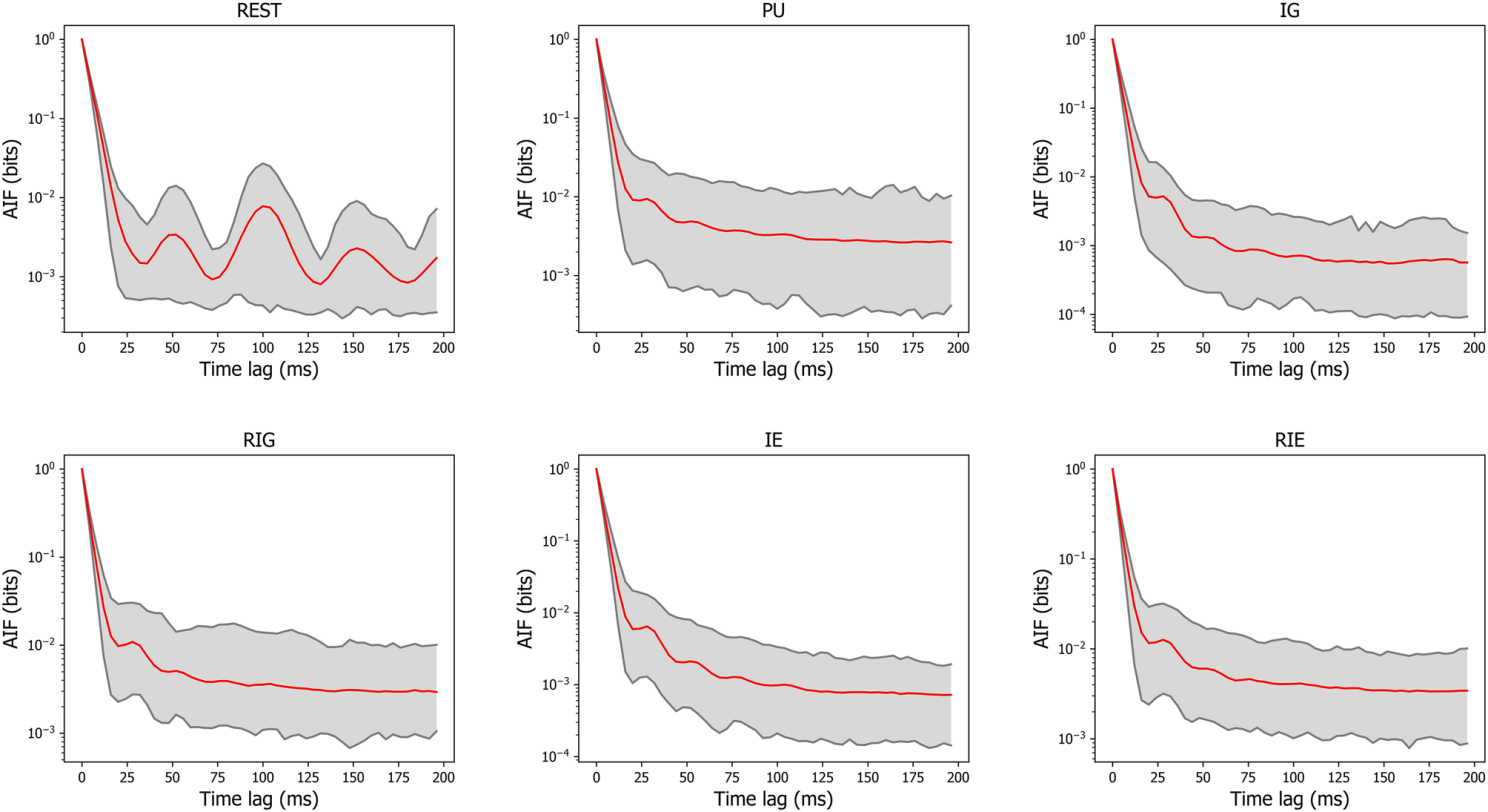
The autoinformation function for each condition. The red line represents the mean autoinformation function across subjects for each condition, while the shaded area represents the 95% confidence interval for each condition.

The first-peak latencies in milliseconds of AIF was 50 (SE = 1.6) for REST, 36 (SE = 1.1) for PU, 42 (SE = 1.6) for IG, 33 (SE = 0.9) for RIG, 36 (SE = 1.1) for IE, and 35 (SE = 1.1) for RIE, when time lags were considered up to 200 ms. Figure 11 shows the mean and 95% confidence interval of AIF for each condition.

**Figure 12:**
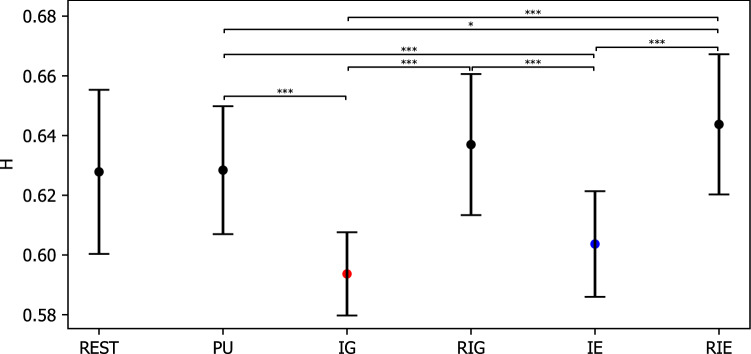
Hurst exponent of microstate sequences averaged from 35 partitions during rest (REST), problem understanding (PU), idea generation (IG), rating idea generation (IE), idea evaluation (IE), and rating idea evaluation (RIE). P-values between rest and other conditions are annotated by black dots ($p>0.050$), blue dots ($p\le 0.050$), yellow dots ($p\le 0.010$), and red dots ($p\le 0.005$). P-values between conditions are annotated by * ($p\le 0.050$), ** ($p\le 0.010$), *** ($p\le 0.005$). Error bars: standard error of the mean.

In addition, the Hurst exponent averaged from 35 partitions was 0.628 (SE = 0.005) for REST, 0.628 (SE = 0.004) for PU, 0.594 (SE = 0.003) for IG, 0.637 (SE = 0.005) for RIG, 0.604 (SE = 0.003) for IE, and 0.644 (SE = 0.005) for RIE. The repeated measures ANOVA revealed a significant effect CONDITION ($F(3.276,85.182)=24.696, p=0.000, \eta ^2=0.487$). Post hoc paired t tests with Bonferroni correction as shown in Fig. 12 revealed that the Hurst exponent was significantly lower during IG and IE compared to during REST, PU, RIG, and RIE ($ps<0.041$), while the Hurst exponent was significantly lower during PU compared to during RIE ($p=0.025$).

## Discussion

Herein, we investigated the temporal dynamics of EEG-defined whole-brain neuronal networks during the conceptual design process in a loosely controlled setting. First, the loosely controlled setting simulated the natural design process to facilitate an ecologically valid neurocognitive study. The experiment setting provides sufficient response time to accommodate the flexibility and freedom necessary for participants to explore potentially creative ideas. Simultaneously, the loosely controlled setting maintained certain degrees of control over the experiment by dividing the experiment into three main sub-design activities, which are problem understanding (PU), idea generation (IG), and idea evaluation (IE). NASA Task Load Index was added after IG and IE, namely rating idea generation (RIG) and rating idea evaluation (RIE), to subjectively measure participants’ mental demand, time demand, performance, effort, and stress level. Secondly, the TRP analysis revealed that IG was associated with significant decreases in delta, theta, alpha, and beta power, suggesting the highest cognitive workload and lowest cognitive control. Finally, the EEG microstate analysis indicated that microstate class C was more prevalent during IG while IG was associated with the shortest correlation times, supporting the lowest cognitive control in IG.

### How are different conceptual design activities associated with cognitive workload?

The alpha band TRP analysis suggests differences among three groups of experimental conditions, which are IG, IE, and PU/RIG/RIE. It was found that IG, IE, and PU/RIG/RIE were associated with decreases in alpha power while the degree of decreases in alpha power was the largest over almost all sites during IG, followed by during IE and PU/RIG/RIE. Decreased alpha power is considered as a reliable indicator of cognitive workload for ecologically valid tasks^65–67^. In addition, decreases in alpha power were associated with task difficulty^68–70^, semantic memory^71,72^, and attention^73^, when these were manipulated to induce different levels of cognitive workload^74,75^. These findings indicated that the IG task triggered the highest cognitive workload, followed by IE and PU/RIG/RIE.

Recent studies in the field of neurocognitive creativity demonstrated the functional role of changes in alpha power. Some studies indicated that alpha power decreased from rest to creativity-related tasks^17,19,68,76^ while others reported that alpha power increased from rest to creativity related tasks following a U-shaped curve^77–80^. The former is in line with the finding that a reduction of alpha power is associated with a more complex and ill-defined problem, especially in ecologically valid settings where a higher cognitive workload is triggered^17,67,68^. In more ecologically valid settings such as design, not only functional but also performance factors need to be considered at the same time. The more factors are considered, the more task-relevant information would be processed and maintained in working memory. Such increased maintenance of task-relevant information is associated with alpha desynchronization^81^. The latter demonstrates an inhibitory top-down control process that inhibits task-irrelevant information to generate creative solutions^82–84^. Therefore, IG would maintain the greatest amount of task-relevant information to generate solutions that meet design requirements, while IE would involve more task-relevant information to make preferential evaluations.

Besides, the obtained NASA-TLX results indicated that mental demand, time demand, effort, and stress decreased significantly from IG to IE, supporting the hypothesis that IG would trigger the highest cognitive workload. The degrees of cognitive workload may be affected by varying task difficulty and complexity, semantic process, and attention. The higher cognitive workload is associated with ill-defined problems compared to well-defined problems^68^, as well as increases in task difficulty^69,70^. Along the same line, the degrees of ill-definedness and task difficulty are the highest during IG, the lowest during PU/RIG/RIE, and at an intermediate level during IE. More specifically, participants are confronted with the most well-defined problems during RIG and RIE, and read/comprehend/decompose design requirements during PU. During IG, on the contrary, participants are dealing with ill-defined problems, synthesising and evaluating knowledge recursively to generate/detail/elaborate solutions, which in turn reformulate design requirements. IE is not as complex or ill-defined as IG, but is more complex than PU/RIG/RIE in that participants apply the knowledge generated in PU and IG to judge the existing solutions without pre-defined judging criteria/constraints. In sum, not only EEG but also behavioural findings suggest that IG would be associated with the highest cognitive workload.

Indeed, cognitive workload and mental stress in conceptual design could be triggered by uncertainty and recursivity, which are two fundamental characteristics of design. Uncertainty inherits from ill-defined design statements and would last throughout the conceptual design process due to recursivity. Incomplete and imprecise information collected from design statements may heighten the degree of uncertainty and unpredictability, which could be linked to mental stress such that Mental Stress = Perceived Workload/((Knowledge + Skills) * Affect) as defined in Nguyen and Zeng^4^. Designer’s affect could be very low when uncertainty becomes high, as Grupe and Nitschke stated that “uncertainty diminishes how efficiently and effectively we can prepare for the future and thus contributes to anxiety”^85^. Consequently, the mental stress would increase and more knowledge and skills are required to compensate for the decreasing affect in designer’s effort to complete the task. Such variations in designer’s mental stress, affect, knowldge, and skills, indicate that cognitive workload may increase with increasing uncertainty, which is supported by a meta-analysis of fMRI studies showing that the brain is more active under conditions of uncertainty, compared to certainty^86^. From this viewpoint, participants tend to experience the most uncertainty during IG whereas they are more certain about their solutions during RIG/RIE. Therefore, the higher cognitive workload would be induced in IG due to its greater degree of uncertainty.

Furthermore, recursivity demonstrates continuous evolution during the conceptual design process in which goals, solutions, and knowledge evolve simultaneously^33–38^. The newly generated solutions will not only improve the designer’s understanding but also help reformulate the design problem. The reformulated design problem will trigger the designer to identify new knowledge to elaborate the previous solutions or regenerate different tentative solutions, which in turn updates the design problem. High cognitive workload would be triggered during such a recursive process as designers need to maintain a large amount of multidimensional information and their relationships with goals, solutions, and knowledge. IG seems to share the most features of the recursive design process whereas PU/RIG/RIE share the most similarity with well-defined problem solving. Therefore, higher cognitive workload would be induced during IG compared to IE and PU/RIG/RIE.

### How are different conceptual design activities associated with cognitive control?

The TRP analysis in delta, theta, and beta bands suggests differences among three groups of experimental conditions, which are IG, IE, and PU/RIG/RIE. It was found that delta, theta, and beta power increased over frontal sites from REST to PU/RIG/RIE and IE, whereas they decreased over all sites from REST to IG. A comparison within design activities indicated that delta, theta, and beta power increased significantly over almost all sites from IG to IE, and PU/RIG/RIE, as well as from IE to PU/RIG/RIE, while delta, theta, and beta power did not show significant differences over almost all sites in PU/RIG/RIE. Increased theta power over the frontal sites has been viewed as a function of working memory and cognitive control. Generally, increased frontal theta activity has been interpreted as a need for increased cognitive control in response to conflict^87,88^, encoding and retrieval of information from working memory^58,89^, while increased beta activity is associated with the maintenance of intended status quo^90^. Besides, increases in delta power have been associated with heightened attention during mental tasks, reflecting the role of inhibiting interferences^91,92^. These findings indicated that IG, IE, PU/RIG/RIE triggered cognitive control with the lowest to the highest intensities, respectively.

Higher cognitive control is beneficial to goal-directed contexts, whereas lower cognitive control is helpful to learning and creative problem-solving contexts^93^. The heightened cognitive control in the PU/RIG/RIE group may result from ignoring distractors in the reading activity, which could improve reading speed and comprehension^94,95^. Alternatively, the increased cognitive control in the PU/RIG/RIE group may result from the “structured” process, which could narrow the focus of attention on a well-defined target. Besides, increases in theta power over frontal sites from IE and RIG/RIE to REST indicated the involvement of more cognitive control during IE and RIG/RIE, which is in line with increases in theta power being associated with heightened cognitive control during the complex decision-making^25^. Interestingly, IE involved less cognitive control compared to RIG/RIE due to the smaller increases in theta power, even if IE and RIG/RIE shared similar evaluative process. This difference in cognitive control may be ascribed to the properties of evaluative criteria, such as abstractness and quantity. Riddle and colleagues reported that a higher level of abstraction rules is linked to decreased beta amplitude while a larger number of rules is associated with increased theta amplitude^28^.

Our analysis indicated the same findings in that beta power decreased significantly over frontal, central, and temporal sites from RIG/RIE to IE while theta power increased significantly over frontal, central, and temporal sites from IE to RIG/RIE. Indeed, IE involved higher level of abstraction rules compared to RIG/RIE in that participants needed to express their preferences without an explicit criterion. IE could be categorized as internally guided decision-making whereas RIG/RIE could be categorized as externally guided decision-making. The internally guided decision-making would involve less cognitive control compared to externally guided decision-making^96^. Similarly, delta power increased significantly from IE to RIG/RIE, suggesting increases in attention during RIG/RIE^91,92^. These findings suggest that higher cognitive control results from the heightened attention during more “structured” processes such as PU/RIG/RIE, while less cognitive control results from internally oriented processes, such as IE.

Furthermore, the lowest cognitive control was associated with IG compared to IE and PU/RIG/RIE. IG is typically viewed as a mixed process between self-generated and task-initiated thoughts^93^. A study of the role of inhibition in creativity revealed that lower cognitive control enhanced the frequency and originality of ideas^97^. In the same vein, lower cognitive control may incubate a few critical activities, such as mind wandering^54^ and hypofrontality^98,99^, to improve creative performance. In addition, a study of musical improvisation indicated that creative improvisation was characterized by a dissociated pattern of activity in the prefrontal cortex^100^. Less activation in the prefrontal cortex could reduce cognitive control, which may help participants overcome fixation or associate objects that are semantically less similar to reinterpret the design problem^101^. In the same lines, neuroimaging studies indicated that creative idea generation is associated with activation of the DMN resulting from reduced cognitive control^10,102^. However, a recent study reported interactions between the DMN and the cognitive control network underlying creativity^12^, suggesting that the balance between the DMN and cognitive control network may benefit flexible regulation for creative performance^103,104^. Our findings regarding decreased alpha and theta power in IG support the argument that IG involves not only increased cognitive workload but also reduced cognitive control. Further study is needed to shed light on the temporal dynamics of brain networks during the conceptual design process.

Interestingly, the right and left hemispheres may play different roles in cognitive control during conceptual design activities. PU was strongly associated with left hemispheric frontal activation in delta, theta, and beta bands, while RIG/RIE was strongly associated with left hemispheric central activation in delta and theta bands. This left hemispherical activation may result from heightened cognitive control being associated with language, judgements, and retrieval processing during PU and RIG/RIE^105,106^. Different from previous findings of right hemisphere dominance in creativity^107^, our results indicated that IG led to different hemispheric specializations over distinct areas. This finding supports that the right-brain theory might not capture all aspects of creative cognition^108^, which should be further investigated.

### How are different conceptual design activities associated with the range of temporal correlations?

Our analysis of microstate parameters and temporal correlations within microstate sequences suggests differences between two groups of experimental conditions, IG and IE on one side, and PU, RIG, RIE on the other side.

Summarizing the microstate parameters coverage, duration, and occurrence, we found a prevalence of microstate classes A and B during the conditions PU/RIG/RIE, whereas microstate classes C and D were more prominent during REST, IG and IE. An increased coverage and occurrence of classes C and D during rest is probably related to the fact that their topography reflects the parieto-occipital dominance of resting-state alpha oscillations. The relation with conditions IG and IE is less clear. One explanation is that all design tasks involved visuo-spatial imagery, which would activate occipital (visual) and parieto-occipital cortices. Combining EEG microstate analysis and source reconstruction, microstate class C has been found to correlate with activity in the precuneus^64^, which is involved in visuospatial processing and introspection, both of which may play a role during IG. In regard to cognitive control, the microstate literature has not reached a consensus so far. As reviewed by Michel and Koenig^42^, positive as well as negative correlations of microstate class C with cognitive control mechanisms have been reported. Assuming that the RIG/RIE conditions correspond to a higher level of cognitive control, our results suggest that microstate class C is negatively correlated with cognitive control, and that microstate classes A and B indicate more control. This interpretation would agree with the results found in the studies of cognitive processes that microstate C reflects activity in the DMN^48,49^.

The microstate classes (E,F,G) showed less pronounced differences between the experimental conditions. Microstate class F was more prominent during IE, and microstate class G was more pronounced during RIG/RIE.

The current literature on the relationship between individual microstate classes and cognitive functions still contains open discussions^42^. Moreover, the assignment of topographies obtained from clustering algorithms to specific microstate classes (A–G) can be challenging, especially when more than four microstate classes are used. For this reason, and hypothesizing that cognitive activities might be better captured by dynamic microstate properties, we analyzed temporal correlations of microstate sequences and found marked differences between our experimental conditions.

We analyzed temporal microstate correlations for short, intermediate and long time scales, and observed the following patterns. Short- and long-range correlations, as measured by the finite entropy rate and Hurst exponents respectively, gave consistent results. The finite entropy rate in IG and IE was significantly larger than in the PU/RIG/RIE group. This indicates a faster decorrelation, or a lower predictability, in the former group. Thus, a short sequence of IG/IE microstates ($k=6$ samples in our case) encodes much less information about which network will activate next, compared to PU/RIE/RIG. A matching observation was made via Hurst exponent analysis for time scales approximately 100 times longer compared to the scale assessed by the entropy rate. Conditions IG and IE showed Hurst exponents closer to $H=0.5$, which indicates uncorrelated activity, and therefore less long-range correlated activity than found in the PU/RIG/RIE conditions. Taken together, these findings suggest that functional brain networks, as measured by EEG microstates, retain less memory about their previous trajectory during IG and IE.

In terms of our cognitive control hypothesis, we conclude that during problem understanding (PU) and rating (RIG,RIE) the brain exerts a stronger cognitive control over network transitions, and that this control is reflected by a more deterministic brain state trajectory, eventually producing a more predictable microstate sequence. During IG and IE, the interplay of functional networks appears less restricted. Interpreting the microstate sequence as a process of stochastic transitions between functional brain networks, a larger entropy rate means that the brain has more degrees of freedom in choosing the next network configuration. In relation to the performed tasks, this less restricted mode of operation might reflect the creativity component of the task, especially during IG, which shows the maximum entropy rate and the lowest Hurst exponent. In our framework, the increasing entropy rate is mediated by a relaxation of cognitive control mechanisms.

In this context, it is interesting to look at intermediate time scales, where oscillatory brain activity becomes apparent. Microstate frequency analysis has been developed only recently, where periodic microstate patterns linked to alpha oscillations were described during the resting state^53^. Our AIF analysis (Fig. 11) shows that the alpha frequency linked microstate oscillations (time lag 100 ms) of the resting state are substituted by higher frequencies as soon as the brain engages in the cognitive tasks. The conditions PU/RIG/RIE show the highest microstate frequencies (AIF peaks at the lowest time lags), corresponding to the lower beta frequency band around 14 Hz. These findings match the TRP analysis where beta and theta frequencies appear during PU/RIG/RIE over bilateral fronto-temporal areas. Likewise, TRP analysis for IG showed a decrease in theta and beta oscillatory activity, explaining why the AIF during IG shows peaks at longer time lags, i.e. less beta frequency contributions. As cognitive control mechanisms are known to be mediated by theta frequencies^89^, we conclude that our cognitive control model is consistent not only with the TRP results, but also with the temporal microstate analysis across all time scales. The added value of microstate frequency analysis is that the identified frequencies indicate periodic behaviour of entire large-scale brain networks, rather than analyzing oscillations at the single sensor level. Our data suggest that cognitive control is associated with periodic activity of large-scale networks in the beta frequency band.

Of note, entropy rates, Hurst exponents and AIF coefficients are independent of how the microstate label assignment to the microstate maps is chosen. As seen in Fig. 6, in the case of seven microstate classes the assignment of the k-means output to the labels A-G reported in the literature can be ambiguous. As the entropy-based quantities (entropy rate, AIF) reported in this study would be the same for any label assignment, this methodology adds further robustness to our results.

### Limitations and future directions

A few limitations of the current study need to be addressed in the future. First, the objective of this present study is to understand the brain activities in problem understanding, idea generation, rating idea generation, idea evaluation, and rating idea evaluation during the conceptual design process. We did not take into account participants’ behaviour data except for NASA-TLX, since some aspects of behavioural analysis heavily rely on subjective criteria. For instance, evaluating design solutions depends on expert knowledge and experience, as well as sample size. Future studies should consider how to analyse participants’ behavioural data in an objective manner and relate them to neurocognitive data. Second, there may be some effects of gender imbalance on brain activities during the conceptual design process^109^. Future studies, including further investigations into the conceptual design process should consider gender-based analyses to avoid possible effects of gender imbalance.

Third, the aim of EEG microstate analysis is to infer the activity of functional brain networks from characteristic EEG topographic patterns. As EEG signals are affected by volume conduction, tissue-dependent signal filtering, and discrete sampling via an electrode array, the relationship between microstates and functional networks is not one-to-one. Thus, functionally different networks may be represented by only one microstate map. Clustering algorithms reliably yield the same four canonical microstates during different cognitive activities^48^, and even across different sleep stages^110^, although network activity during sleep is likely to be different from task execution, especially in regard to the cognitive control mechanisms discussed in this paper. Therefore, the approach of identifying cognitive processes with individual microstate maps is probably too simple, and can be the source of contradicting findings^42^. Some aspects of task execution may be better characterized by the dynamic sequence in which networks are activated, rather than by the averaged prevalence or duration of a specific microstate map. We have tried to overcome these limitations by adding dynamic and label-independent measures (entropy rate, AIF, Hurst exponent) and we believe that the congruence with the results from TRP analysis corroborates our interpretations. To further clarify the networks involved in design task execution, future studies may benefit from methods such as high-density EEG combined with source localization, or functional MR imaging approaches, although the latter imposes restrictions on the experimental setting.

## Conclusions

This present study was designed to investigate temporal dynamics of brain activity in response to distinct design activities during the conceptual design process through a loosely controlled setting. The loosely controlled setting simulated the natural design process to facilitate an ecologically valid neurocognitive study, which offered sufficient response time for participants to freely explore potentially creative solutions while maintaining certain degrees of control through segmenting the conceptual design process into sub-design activities, including problem understanding, idea generation, rating idea generation, idea evaluation, and rating idea evaluation. Aligning our findings with those of other validated evidence, the TRP analysis revealed that idea generation was associated with significant decreases in delta, theta, alpha, and beta power, suggesting the highest cognitive workload and lowest cognitive control. In the same vein, the EEG microstate analysis indicated that microstate class C was more prominent during idea generation. Further temporal dynamics analysis found that idea generation was consistently associated with the shortest correlation times, as measured by the finite entropy rate, AIF, and Hurst exponent. This finding suggests that the interplay of functional brain networks is less restricted during idea generation, supporting the idea that the brain has more degrees of freedom during tasks involving creativity. Taken together, we conclude that idea generation is associated with the highest cognitive workload and lowest cognitive control, consistently supported by TRP and microstate analysis.

## Data Availability

The data set analysed in the current study and code of TRP and EEG microstate analysis are available in the G-Node repository as follows: https://gin.g-node.org/Design-Lab/Network-oscillations-in-open-ended-creation-tasks.

## Code availability

The codes used for TRP and EEG microstate analysis are made publicly available in the G-Node repository as follows: https://gin.g-node.org/Design-Lab/Network-oscillations-in-open-ended-creation-tasks/src/master/code.
